# A comparison of spatial heterogeneity with local cluster detection methods for chronic respiratory diseases in Thailand

**DOI:** 10.12688/f1000research.12128.2

**Published:** 2018-03-06

**Authors:** Wongsa Laohasiriwong, Nattapong Puttanapong, Amornrat Luenam

**Affiliations:** 1 Faculty of Public Health and Research and Training Center for Enhancing Quality of Life for Working Age People, Khon Kaen University, Khon Kaen, Thailand; 2 Faculty of Economics, Thammasat University, Khlong Luang, Pathum Thani, Thailand; 3 Faculty of Public Health, Khon Kaen University, Khon Kaen, Thailand

**Keywords:** chronic respiratory diseases, comparison, spatial heterogeneity, local cluster detection, methods, Thailand

## Abstract

**Background**: The Centers for Disease Control and Prevention reported that deaths from chronic respiratory diseases (CRDs) in Thailand increased by almost 13% in 2010, along with an increased burden related to the disease. Evaluating the geographical heterogeneity of CRDs is important for surveillance. Previous studies have indicated that socioeconomic status has an effect on disease, and that this can be measured with variables such as night-time lights (NTLs) and industrial density (ID). However, there is no understanding of how NTLs and ID correlate with CRDs. We compared spatial heterogeneity obtained by using local cluster detection methods for CRDs and by correlating NTLs and ID with CRDs.

**Methods**: We applied the spatial scan statistic in SaTScan, as well as local indices of spatial association (LISA), Getis and Ord’s local Gi*(d) statistic, and Pearson correlation. In our analysis, data were collected on gender, age, household income, education, family size, occupation, region, residential area, housing construction materials, cooking fuels, smoking status and previously diagnosed CRDs by a physician from the National Socioeconomic Survey, which is a cross-sectional study conducted by the National Statistical Office of Thailand in 2010.

**Results**: According to our findings, the spatial scan statistic, LISA, and the local Gi*(d) statistic revealed similar results for areas with the highest clustering of CRDs. However, the hotspots for the spatial scan statistic covered a wider area than LISA and the local Gi*(d) statistic. In addition, there were persistent hotspots in Bangkok and the perimeter provinces. NTLs and ID have a positive correlation with CRDs.

**Conclusions**: This study demonstrates that all the statistical methods used could detect spatial heterogeneity of CRDs. NTLs and ID can serve as new parameters for determining disease hotspots by representing the population and industrial boom that typically contributes to epidemics.

## Introduction

Chronic respiratory diseases (CRDs) are a public health issue worldwide ^1^. According to the World Health Organization, CRDs are the world’s leading cause of death. In Thailand, the Centers for Disease Control and Prevention reported that deaths from CRDs increased by almost 13% in 2010, and there was an increased burden resulting from these diseases ^2^.

Spatial clustering methods are important for statistical consideration, to develop models for prediction of disease risk positions. Disease risk positions are areas located near one another in statistically significant clusters. In this study, we indicate whether the geographical aggregation of CRDs is explained by chance or is statistically significant. It is likely that geographical areas share similar disease risk factors, since they are in a similar environment. Local spatial clustering methods are useful to identify the characteristics of clusters in terms of location, size, and prevalence of the disease ^3^. These methods differ from standard methods ^3, 4^ used to identify the presence of spatial clustering in a whole study area. Local clustering methods in spatial epidemiology that are commonly applied to understanding spatial clustering (heterogeneity) are the Getis and Ord’s local Gi*(d) statistic, spatial scan statistics, and local indices of spatial association (LISA) ^5– 8^.

At present, there are no studies comparing the performance of these methods in epidemiological studies involving CRDs. In this study, we used local clustering methods to detect the spatial heterogeneity of CRDs and compared the performance of these local spatial clustering methods, including the local Gi*(d) statistic, the spatial scan statistic, and LISA. Spatial scan statistics performed in SaTScan circular version (version 9.4.2) were considered the best choice for detecting small, compact clusters ^9^. A local version of Moran’s I was one of our selective cluster detection methods, due to its great performance on outlier detection, despite not being a good method for detecting large clusters ^3, 10^. The local Gi*(d) statistic was used to indicate locations surrounded by a cluster of high or low values. It shows areas where lower than average values tend to be found near each other, or areas where higher than average values tend to be found near each other, including the value at the location in which the spatial autocorrelation is being measured.

In a socioeconomic context, previous literature has recommended that night-time lights (NTLs) can substitute for other variables, such as urbanization, density, and economic growth. Data on NTLs have been used to indicate the actual scope of urban incorporations and appraise population scale in urban areas ^11– 15^ and population density ^16^. These data have been employed to monitor the speed of urbanization ^17, 18^, as well as electricity used ^19^. Moreover, NTLs have been utilized as a substitute for Gross Domestic Product (GDP), which works as an indirect indicator of administrative areas ^11, 20– 24^. Remotely sensed NTL data can also be applied in epidemiological studies ^25, 26^. A 2011 study entitled “Explaining seasonal fluctuations of measles in Niger using night-time lights imagery” in Niger, by Bharti *et al.,* found the concentration of the population affected by outbreaks of measles, and recommended that NTLs should be applied to public health studies ^27^. Some studies in Israel have found that the incidence of breast and lung cancer in females is associated with NTL intensity in Israel ^28^.

In Thailand, NTLs are used to estimate Gross Provincial Products (GPPs), because the luminosity of NTLs is normally dependent on the amount of economic activity in each area. The results showed that NTLs and GPP growth are significantly correlated and can represent the relationship between economic values and spatial inequality ^29^. Industrial density (ID) may also be related to disease. However, previous studies have not investigated how NTLs and ID correlate with CRDs.

The government of Thailand has utilized various measures to attempt to prevent, diagnose, and treat CRDs. However, spatial clustering of diseases, particularly at a national level, has rarely been applied to examine characteristics of CRD clusters in terms of their location, size, and magnitude in Thailand. In addition, there has been limited use of socioeconomic indicators, such as NTLs and ID, in the epidemiology of CRDs.

In this study, commonly used methods for spatial clustering of CRDs were compared, including the use of the spatial scan statistic, LISA, and the local Gi*(d) statistic. We also analysed whether there was a correlation between NTLs and ID with CRDs. Our findings could help decision makers implement more relevant disease control policies using the performance of these local cluster detection methods, as well as the correlation of CRDs with NTLs and ID. The availability of a suitable strategy for spatial clustering of CRDs may allow for a better allocation of health care resources.

## Methods

### Study area

Thailand occupies an area of 514,000 square kilometres, which consists of an area of 511,770 square kilometres of land and 2,230 square kilometres of water. Thailand shares a border with Myanmar, Cambodia, Laos and Malaysia. In 2010, there were 76 provinces, 878 districts (Amphoe), 7,225 sub-districts (Tambon), and 74,965 villages (Mooban) in Thailand.

### Ethical approval

The Ethical Committee of Khon Kaen University deemed this study to be exempt from requiring ethical approval (reference no. HE 582315).

### Data collection and processing

***Spatial data.*** The geographical coordinates of administrative areas of Thailand were retrieved from DIVA-GIS online ( http://www.diva-gis.org/gdata), which is publicly available. This database of provincial maps of Thailand was processed with Quantum GIS for further GIS based analysis. For GIS based analysis, we applied zonal statistics to calculate mean NTLs and ID. The spatial distribution of CRD prevalence was obtained from the province-level polygon map, which contains information regarding the latitudes and longitudes of each province. The calculated mean NTLs, ID and CRD prevalence were further visualized with province-level layers on the polygon map and labelled with the administrative code of each province.

***CRD, NTL and ID data.*** This study used data from the National Socioeconomics Survey (NSS), a cross-sectional study conducted by the National Statistical Office (NSO), Thailand, in 2010. Using stratified two-stage sampling, the survey selected a nationally representative sample to respond to a structured questionnaire. The questionnaires collected information on gender, age, household income, education, family size, occupation, region, residential area, housing construction materials, cooking fuels, smoking status and previous diagnosis of CRDs. A total of 17,040 individuals who met the inclusion criteria (aged between 18–59 years and diagnosed with having CRDs by a physician) were included in this analysis. The NSO administrative board officially allowed the research team to use the data (reference no.050601/1441).

The NTLs of Thailand used in this study were based on global stable lights imagery from 2010, acquired from the Operational Linescan System (OLS) sensor on-board satellite F18 under the Defense Meteorological Satellite Program (DMSP). All NTL data are publicly available ( https://www.ngdc.noaa.gov/eog/dmsp/downloadV4composites.html). The ID data were calculated from GPPs, which represent the official statistics on provincial economic activity published by the National Economic and Social Development Board (NESDB). GPP data are also publicly available ( http://www.nesdb.go.th/nesdb_en/more_news.php?cid=156&filename=index).

### Local spatial pattern detection methods

The Open GIS software Quantum GIS (version 2.8.5) was used to conduct exploratory spatial data analysis ^30^. Spatial autocorrelation analysis was conducted using GeoDa (version 1.6.6) and the local Gi*(d) statistic ^31^. Stata version 10.0 (StataCorp, CollegeStation, TX) was used to calculate CRD prevalence. SaTScan (version 9.0.1) was used to determine the presence of statistically significant CRD spatial clusters and identify their approximate locations.

***Local indices of spatial association.*** The local Moran’s I investigates the local level of spatial autocorrelation of provinces with a high and low prevalence of CRDs ^32^. Computation of LISA assesses the local version of Moran’s I for each location to determine the variation in spatial autocorrelation over the study area. Its significance is evaluated in five categories: High-High, Low-Low, Low-High, High-Low, and Not Significant. Spatial autocorrelation occurs when a high prevalence of CRDs correlates with a high prevalence in neighbouring areas (also known as hotspots), or when a low prevalence of CRDs correlates with a low prevalence in neighbouring areas (also known as cold spots) ^33^. This study set the spatial weight matrix of 3 k-Nearest Neighbours around each province, meaning that the clustering relation was identified with three neighbouring provinces. The statistical significance level was 0.05, and the simulation used 999 permutations ^34^ to evaluate the sensitivity of the results.

Getis and Ord’s local Gi*(d) statistic. The local Gi*(d) statistic is used to test the statistical significance of local clusters and to determine the spatial dependence and relative magnitude between observations. In this study, the local Gi*(d) statistic was used to test statistically significant CRD prevalence and local autocorrelation, as well as to determine the dependence of neighbouring observation ^35^ and local clustering. We used k-Nearest Neighbours, and contiguity was set as three provinces for a polygon contiguity spatial weight matrix, which was created depending on three provinces that share common boundaries and vertices. A 0.05 significance level and 999 permutations were used to identify significant clusters of local autocorrelation. A high value for the local Gi*(d) statistic indicates a hotspot, and a low value indicates a cold spot ^36, 37^.

***Spatial scan statistic.*** The spatial scan statistic, calculated in SaTScan ^38^, was applied to analyse the geographic distribution of CRD prevalence in Thailand and determine whether there are any spatial geographical clusters of CRD prevalence as for high or low CRDs. We used the retrospective purely spatial Poisson model to identify spatially high or low clusters of CRDs, since it fit the assumption that the number of events in an area was Poisson distributed according to a known underlying population at risk. Purely spatial analysis, which ignores the time dimension of the cases, was performed to detect CRD clusters in the study areas. The purely spatial Poisson model imposed circular windows on the map and let the circle move over the area. Each circle moved until reaching a maximum population at risk. The radius of each circle increased continuously from to a maximum radius. Therefore, the window never encompassed more than 50% of the total population at risk, i.e., the maximum spatial cluster size, in this investigation. The likelihood function was maximized over all window positions and sizes, and the one with the maximum likelihood constituted the most likely cluster. For each window, we used a Monte Carlo simulation to test the null hypothesis that no significant clusters were created, supposing that the relative risk (RR) of CRDs was no different within the window compared to outside the window. The maximum number of random Monte Carlo replications was defined as 999. The likelihood function was maximized in the overall window locations and sizes ^39^. The window with the maximum likelihood value of the spatial window was set to 50% of the population at risk.

## Results

**Figure 1.**
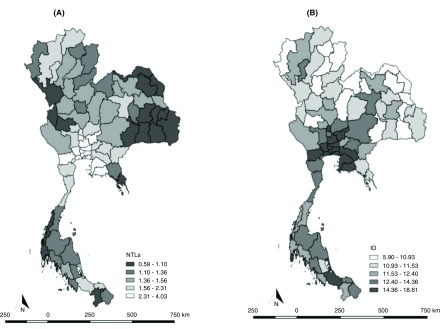
Night-time lights (NTLs) and industrial density (ID) maps for Thailand, 2010. ( **A**) NTLs and ( **B**) ID showed high values, representative of high population density.

Pearson correlation assessed the relationship between NTLs and ID with CRDs in all provinces. There was a moderate positive correlation of 0.34 (95% CI: 0.12-0.52; p-value 0.002) and 0.36 (95% CI: 0.15-0.54; p-value 0.001) with CRDs, which indicated that NTLs and ID had similar relationships to CRDs ( Table 1 and Figure 1).

**Table 1.** Pearson correlation of NTLs and ID with CRDs, and 95% confidence interval (CI) for each factor in 2010.

Factors	CRDs	95% CI	p-value
NTLs	0.34	0.12-0.52	0.002
ID	0.36	0.15-0.54	0.001

The Univariate Moran’s I scatter of annual CRD prevalence among provinces in Thailand in 2010 showed a slightly positive spatial autocorrelation, as the Moran’s I was 0.072, with statistical significance at 0.05. The Moran’s I indicated clustering patterns. There were hotspots in Bangkok and the perimeter provinces, namely, Samut Prakan, Nonthaburi, Pathum Thani and Nakhon Pathom. Cold spots and low prevalence areas, were located in the Nan and Phitsanulok provinces. Two high/low clusters were located in Trang, Songkhla, and part of the Phatthalung provinces, indicating that the distribution of the CRD in affected provinces was spatially autocorrelated (low clustering), even though the overall tendencies were not clear. This could be due to the nature of local Moran’s I indices; the statistic cannot accommodate extreme values or outliers in the data set, such as the very low CRD prevalence in some provinces that were also included in the model. Therefore, its sensitivity in cluster detection may be reduced. Using results from other local spatial analysis methods could help explain the autocorrelations.

Considering NTLs and ID with CRDs in terms of bivariate Moran’s I scatter for annualized prevalence in 2010, the results showed a positive spatial autocorrelation, with Moran’s I values of 0.345 and 0.374, respectively. These values were statistically significant, with a significance level of 0.05. Based on these results, hotspots were located in Bangkok and perimeter provinces, consisting of Samut Prakan, Nonthaburi, Pathum Thani and Nakhon Pathom. The results showed a high, significant correlation between the NTLs with CRDs, suggesting that brighter lights, together with high population densities of exclusively wealthier people, can be used to define the spatial extent of CRD development. Therefore, ID, which reflects urbanization, was correlated with CRDs ( Figure 3 and Figure 4).

For the local Gi*(d) statistics, hotspots were located in Bangkok and perimeter provinces, consisting of Samut Prakan, Nonthaburi, Pathum Thani and Nakhon Pathom ( Figure 5).

**Figure 2.**
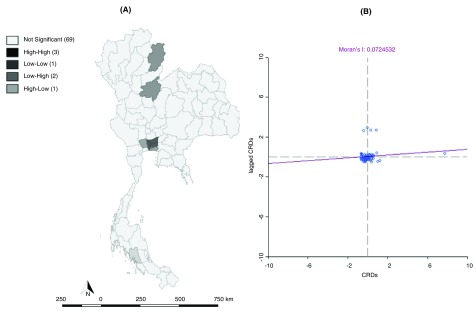
( **A**) LISA (Univariate) cluster map, and ( **B**) Moran scatter plot matrix of CRDs (p < 0.05) in 2010. There were 3 hotspots located in Bangkok and the perimeter provinces.

**Figure 3.**
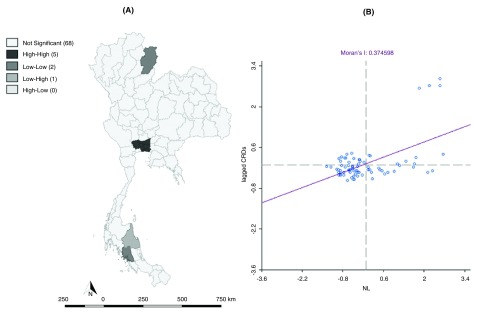
( **A**) LISA (Bivariate; Night-time light with CRDs) cluster map, and ( **B**) Moran scatter plot matrix of CRDs (p < 0.05) in 2010. We analysed NTLs with CRDs in terms of bivariate Moran’s I scatter for annualized prevalence. There were 5 hotspots located in Bangkok and the perimeter provinces.

**Figure 4.**
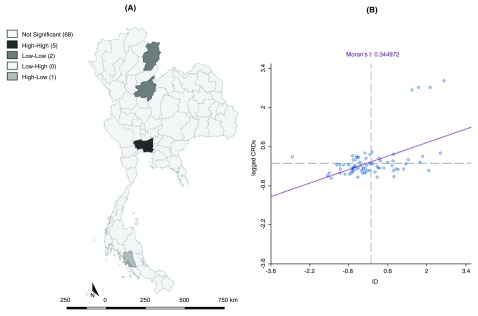
( **A**) LISA (bivariate; industrial density with CRDs) cluster map, and ( **B**) Moran’s I scatter plot matrix of CRDs (p < 0.05) in 2010. We analysed ID with CRDs in terms of bivariate Moran’s I scatter for annualized prevalence. There were 5 hotspots located in Bangkok and the perimeter provinces.

**Figure 5.**
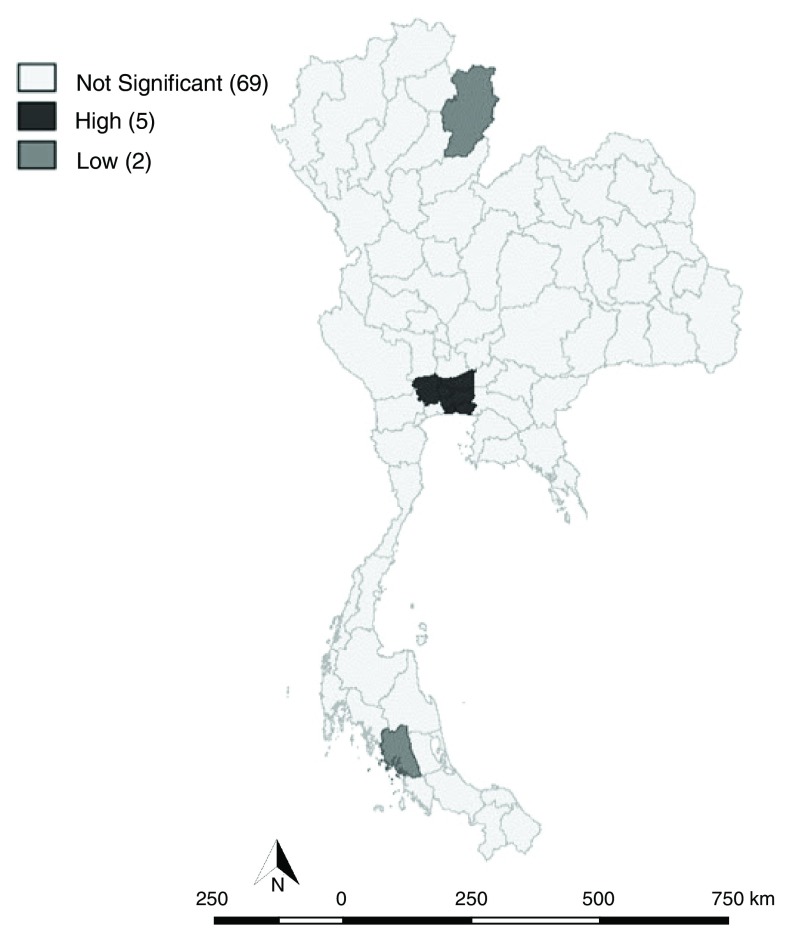
Local Gi*(d) Cluster map of CRDs in 2010. There were 5 hotspots located in Bangkok and the perimeter provinces.

**Figure 6.**
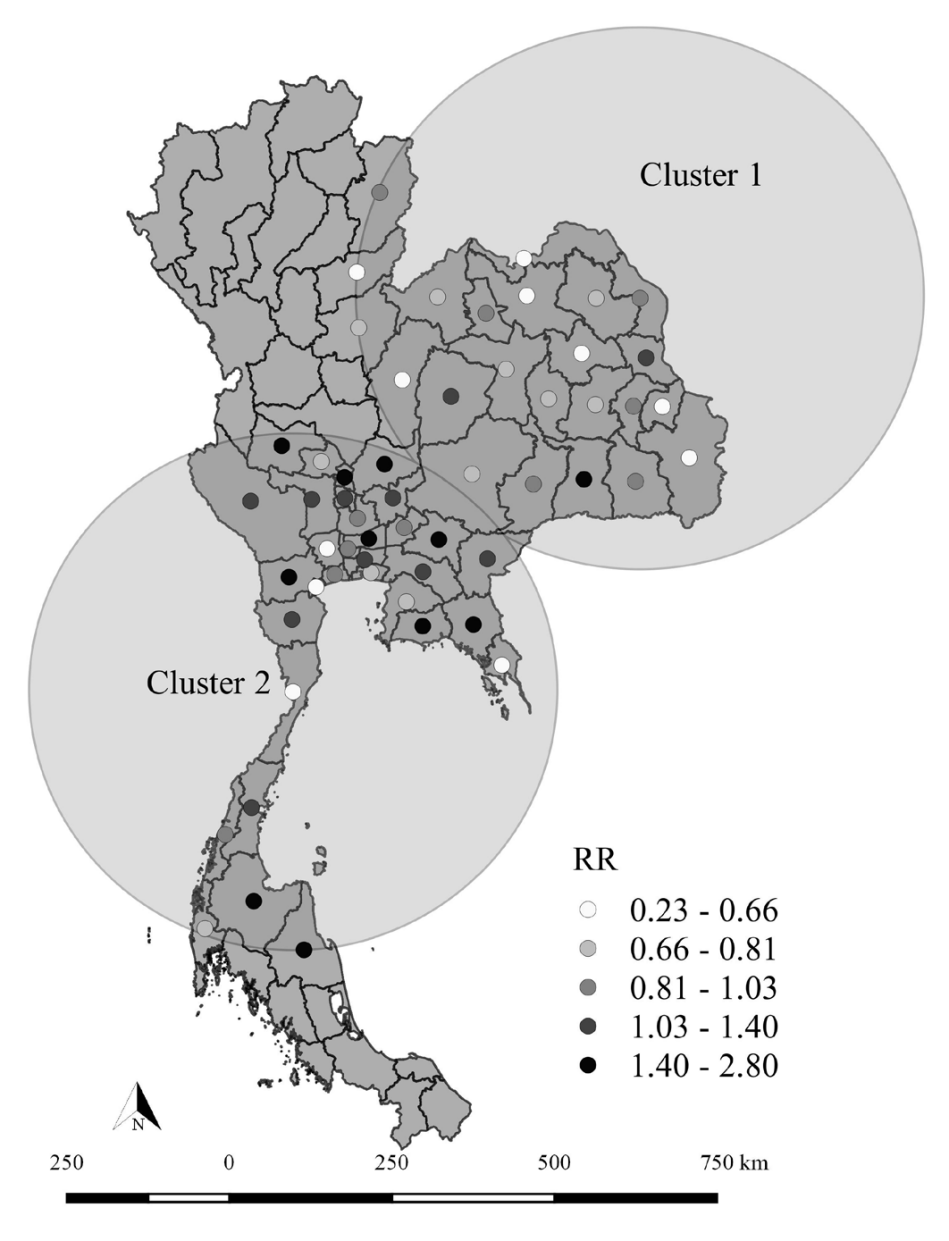
Spatial scan statistic calculated in SaTScan, to detect CRD prevalence in 2010. The spatial scan statistic, calculated in SaTScan, was used to detect spatial high or low CRD prevalence. We used the retrospective, purely spatial Poisson model. Based on these results, the spatial scan statistics revealed two spatial clusters.

 The spatial scan statistics revealed two spatial clusters. The primary cluster was located in 23 provinces. There were 157 cases compared to 213 expected cases. Thus, the ratio between observed and expected cases was 0.74. The p-value was smaller than 0.05, which indicated that the cluster was highly significant. The relative risk for the population inside the cluster compared to the population outside the cluster was 0.65, indicating that the risk of CRDs within this area was higher than locations outside it. This number is the estimated risk within the cluster, divided by the estimated risk outside the cluster. The analysis also revealed that a secondary cluster, which is the most likely cluster, was located in 32 provinces. The number of observed cases was 348, compared to 297 expected cases. Thus, the ratio between observed and expected cases was 1.17. According to these calculations, the relative risk inside the cluster is 1.37. The p-value was smaller than 0.05, indicating that the cluster was highly significant. The relative risk for the population inside the cluster compared to the population outside the cluster was 1.37, indicating that the risk of CRDs in this area was higher than locations outside it ( Figure 6).

 In summary, as seen in Figure 1– Figure 6, spatial scan statistics, LISA, and local Gi*(d) statistics revealed similar results for areas with the highest clustering of CRDs. Nevertheless, the clusters for spatial scan statistics covered a wider area of the Bangkok, Central, Northeast and South clusters, while the clusters for LISA and the local Gi*(d) statistic were wider and more dispersed in Bangkok and the perimeter provinces.

## Discussion

### Comparison of the local spatial pattern detection methods

In this study, NTL and ID data were utilized to assess growth in provinces based on geography, economic growth, urbanization, or health metrics, as well as concentration of NTLs and ID. Areas with a high CRD prevalence also experienced high light growth and a high concentration of ID, and therefore NTLs and ID were probably induced CRDs. In other words, the results indicated that CRDs are affected by population density and industrial concentration. This finding was consistent with a previous study that stated that it is possible to use NTLs in public health studies ^26^. The findings of this study suggest that NTLs and ID can substitute for some variables, such as urbanization, density, economic growth, and industrial concentration. NTLs and ID serve as a new tool for specifying disease hotspots by representing the population and industrial booms typically contributing to epidemics. In urban areas with migration, NTLs can indicate where populations are clustering, through the captured expansion and the increase brightness of lighted areas. The technique indicates fluctuations in population density, which affect epidemic risk and can replace current methods of outbreak surveillance ^26, 40^.

LISA, spatial scan statistics, and local Gi*(d) statistics are common methods applied to investigate local clusters of diseases. These techniques help identify clusters and assess their statistical significance ^41^. In this study, all of these methods provided comparable results in the detection of geographic areas of CRDs in both high and low- rate clusters. However, there were some inconsistencies. The identification of clusters by spatial scan statistics was more localized, compared to LISA and local Gi*(d) statistics, possibly due to their dissimilar role in defining clusters. LISA detected the neighbouring provinces with rates significantly correlated to each other, and areas with similar values were thus determined as a cluster ^32^. In this study, LISA explored the correlation between the value of a certain area and the average value of neighbouring areas. Spatial scan statistic methods were used, and larger clusters were detected; the calculation of the maximum likelihood ratio of cases was made together with the underlying population in the area to investigate the cluster of provinces considered to have higher or lower rates. Using spatial scan statistics methods, larger clusters were detected, and the calculation of maximum likelihood ratio of cases was made simultaneously with the underlying population in the area to investigate the cluster of provinces and consider them for higher or lower rates ^7, 42^. Meanwhile, the local Gi*(d) statistic and LISA reported that the two methods shared similar results in terms of rising clusters (hotspots). Hanson and Wieczorek ^7^ compared LISA to spatial scan statistics by exploring alcohol-related mortality in New York, USA (which identified some differences between two methods). It was concluded that the spatial scan statistic is the more sensitive method. A multi-method approach is adopted for cluster detection, since different methods tend to identify different characteristics in clusters, i.e., LISA identifies the core of the cluster, and the scan statistic identifies its extent. Furthermore, Jacquez and Greiling ^43^ used LISA to analyse spatial clustering for diagnosis of breast, lung and colorectal cancers in Long Island, USA, and to identify significant spatial patterns for all of these diseases. Their analysis confirms the clustering of breast cancer mortality previously revealed by Kulldorff’s spatial scan statistic ^44^, but they found that the two methods identified slightly different clusters. As a result, they recommended that a combination of statistics be used when studying local clustering to assure that different aspects of spatial patterns are fully identified and that the results from the suite of analyses are logically consistent.

Due to its own principle for cluster detection, each method selected different geographic areas. The intersection of the selections indicated that these three methods resulted in different features in the same clusters, suggesting that various methods should be applied to identify clusters of CRDs at the provincial level in Thailand. These spatial cluster detection methods can be utilized collectively rather than individually. Furthermore, because each method has its own advantages and disadvantages, no single method is deemed the “gold standard” for cluster analysis ^45^.

We study the CRDs morbidity which is mainly linked to the epidemic of tobacco exposure and indoor and outdoor air pollution in Asian countries ^46^, whereas the mortality may relate with the accessibility to healthcare service in Bangkok metropolitan and some surrounding area.

The strengths of this study included the methods used to analyse CRD cases, including spatial scan statistics, LISA, and local Gi*(d) statistics. All of these have been thoroughly investigated and compared to determine the appropriate methods for evaluating spatial clustering and cluster detection. NTLs and ID were included to investigate a correlation with CRDs. This study contained a large sample size that represents a nationally representative sample. Therefore, the results could represent the Thai population in general.

### Limitations

There are two aspects of limitations. The first is based on the availability of data. Therefore, there are limited resolutions in both spatial and time dimensions of data. The second limitation is caused by the characteristic of data and analytical method that do not permit the cause-and-effect conclusion, particularly the connection between individual exposure histories and individual outcome events.

**Abbreviations:** CRDs: Chronic respiratory diseases, NSO: National Statistical Office, NSS: National Socioeconomics Survey, LISA: Local Indices of Spatial Association, ID: Industrial density, NTLs: Night time lights.

Pearson correlation with CRDs dataset Click here for additional data file. Copyright: © 2018 Laohasiriwong W et al. 2018 Data associated with the article are available under the terms of the Creative Commons Zero "No rights reserved" data waiver (CC0 1.0 Public domain dedication).

NTLs and ID dataset Click here for additional data file. Copyright: © 2018 Laohasiriwong W et al. 2018 Data associated with the article are available under the terms of the Creative Commons Zero "No rights reserved" data waiver (CC0 1.0 Public domain dedication).

LISA (Univariate) cluster CRDs dataset Click here for additional data file. Copyright: © 2018 Laohasiriwong W et al. 2018 Data associated with the article are available under the terms of the Creative Commons Zero "No rights reserved" data waiver (CC0 1.0 Public domain dedication).

LISA (Bivariate; Night-Time Light with CRDs) cluster dataset Click here for additional data file. Copyright: © 2018 Laohasiriwong W et al. 2018 Data associated with the article are available under the terms of the Creative Commons Zero "No rights reserved" data waiver (CC0 1.0 Public domain dedication).

LISA (Bivariate; Industrial Density with CRDs) cluster dataset Click here for additional data file. Copyright: © 2018 Laohasiriwong W et al. 2018 Data associated with the article are available under the terms of the Creative Commons Zero "No rights reserved" data waiver (CC0 1.0 Public domain dedication).

Local Gi*(d) cluster CRDs dataset Click here for additional data file. Copyright: © 2018 Laohasiriwong W et al. 2018 Data associated with the article are available under the terms of the Creative Commons Zero "No rights reserved" data waiver (CC0 1.0 Public domain dedication).

Spatial scan statistics in SaTScan analysis of CRDs dataset Click here for additional data file. Copyright: © 2018 Laohasiriwong W et al. 2018 Data associated with the article are available under the terms of the Creative Commons Zero "No rights reserved" data waiver (CC0 1.0 Public domain dedication).

## Conclusions

This study utilized a geographic information system and spatial analyses, which can be applied to several epidemiological studies to investigate and clarify the spatial heterogeneity of CRDs in highly affected provinces in Thailand. LISA, spatial scan statistics, and local Gi*(d) statistics were calculated, with the aim of revealing the spatial characteristics of the CRDs. Spatial scan statistics performed much better in outlier detection in terms of power, compared to LISA and local Gi*(d) statistics. Based on our simulation, there was a large relative risk difference (two spatial clusters) and significant spatial heterogeneity. The findings suggested heterogeneity in the spatial pattern of CRDs in the study. Purely spatial retrospective analysis revealed persistence of CRD clusters in some geographical locations of the provinces on an annual basis. CRDs were affected by the concentration of NTLs and ID. We believe that NTLs and ID serve as a new tool for specifying disease hotspots by representing the population and industrial booms that typically contribute to epidemics. The results of the study provide helpful information on the common epidemiological situation of CRDs. Demonstrating the existence of CRDs hotspots in different provinces may enable the Ministry of Public Health or provincial health officers to launch remedial measures in the affected areas and formulate strategies for more effective handling of CRDs.

## Data availability

The data referenced by this article are under copyright with the following copyright statement: Copyright: © 2018 Laohasiriwong W et al.

Data associated with the article are available under the terms of the Creative Commons Zero "No rights reserved" data waiver (CC0 1.0 Public domain dedication). 

Data used in this study were from the NSS; permission to use these data can be requested from the NSO. This study got approval from the NSS (reference no.050601/1441) to use the data on gender, age, household income, education, family size, occupation, region, residential area, housing construction materials, cooking fuels, smoking status and previously diagnosed CRDs by a physician. NTL data and ID data are publicly available.

The NTL data are from the Operational Linescan System (OLS) sensor on-board satellite F18 under the Defense Meteorological Satellite Program (DMSP) ( https://www.ngdc.noaa.gov/eog/dmsp/downloadV4composites.html).

The ID data are from the National Economic and Social Development Board (NESDB) ( http://www.nesdb.go.th/nesdb_en/more_news.php?cid=156&filename=index).

Dataset 1. Pearson correlation with CRDs dataset.

DOI, 10.5256/f1000research.12128.d178700 ^47^

Dataset 2. NTLs and ID dataset.

DOI, 10.5256/f1000research.12128.d178701 ^48^

Dataset 3. LISA (Univariate) cluster CRDs dataset.

DOI, 10.5256/f1000research.12128.d178702 ^49^

Dataset 4. LISA (Bivariate; Night-Time Light with CRDs) cluster dataset.

DOI, 10.5256/f1000research.12128.d178703 ^50^

Dataset 5. LISA (Bivariate; Industrial Density with CRDs) cluster dataset.

DOI, 10.5256/f1000research.12128.d178704 ^51^

Dataset 6. Local Gi*(d) cluster CRDs dataset.

DOI, 10.5256/f1000research.12128.d178705 ^52^

Dataset 7. Spatial scan statistics in SaTScan analysis of CRDs dataset.

DOI, 10.5256/f1000research.12128.d178706 ^53^
