# Supplementary material for: A comparison of spatial heterogeneity with local cluster detection methods for chronic respiratory diseases in Thailand
Source: F1000Res. 2018 Mar 6;6:1819. Originally published 2017 Oct 9. [Version 2] doi: 10.12688/f1000research.12128.2 (PMC5874503; doi:10.12688/f1000research.12128.2)
Supplement: Peer review history

# Peer review: Referee response for version 2

**DOI:** 10.5256/f1000research.15403.r31532

**Published:** 2018-03-28

**Authors:** Mathuros Tipayamongkholgul

**Affiliations:** 1 Department of Epidemiology, Faculty of Public Health, Mahidol University, Bangkok, Thailand

I have read this submission. I believe that I have an appropriate level of expertise to confirm that it is of an acceptable scientific standard.

# Peer review: Referee response for version 2

**DOI:** 10.5256/f1000research.15403.r31533

**Published:** 2018-03-06

**Authors:** Edson Zangiacomi Martinez

**Affiliations:** 1 Ribeirao Preto Medical School, University of Sao Paulo, Monte Alegre, Brazil

All suggestions were incorporated in the revised version and I recommend that the article be published in F1000Research.

I have read this submission. I believe that I have an appropriate level of expertise to confirm that it is of an acceptable scientific standard.

# Peer review: Referee response for version 1

**DOI:** 10.5256/f1000research.13126.r29646

**Published:** 2018-01-29

**Authors:** Edson Zangiacomi Martinez

**Affiliations:** 1 Ribeirao Preto Medical School, University of Sao Paulo, Monte Alegre, Brazil

The authors present interesting data about the possible associations between socioeconomic status (night-time lights and industrial density) and chronic respiratory diseases. The manuscript is well written,  the methods and the results sections are adequate, and should be of great interest to the readers. Overall, it is an important study, but their limitations are not mentioned. For example, ecological studies do not permit cause-and-effect conclusions or evidence that risk-modifying factors are related to risk of the disease *, *since they do not link individual exposure histories to individual outcome events. Thus, I believe the authors can provide a paragraph on the limitations of the study.

I have read this submission. I believe that I have an appropriate level of expertise to confirm that it is of an acceptable scientific standard, however I have significant reservations, as outlined above.

# Response

**Published:** 2018-02-28

**Authors:** Amornrat Luenam

**Affiliations:** Faculty of Public Health, Khon Kaen University, Thailand

There are two aspects of limitations. The first is based on the availability of data. Therefore, there are limited resolutions in both spatial and time dimensions of data. The second limitation is caused by the characteristic of data and analytical method that do not permit the cause-and-effect conclusion, particularly the connection between individual exposure histories and individual outcome events.

# Peer review: Referee response for version 1

**DOI:** 10.5256/f1000research.13126.r28274

**Published:** 2017-12-18

**Authors:** Mathuros Tipayamongkholgul

**Affiliations:** 1 Department of Epidemiology, Faculty of Public Health, Mahidol University, Bangkok, Thailand

1. In the methods, the author used SatScan and used 50% of population at risk to be a radius at the province level. Using such technique at the provincial level (large geographic area) may lead to inaccurate cluster as you can see in the result that one cluster cover the whole region.
2. In the result, Moran’ I present very low spatial correlation (0.07), although it presented statistically significant. The correlational value cannot accept the spatial correlation that exists, and therefore all following spatial data analysis may not be necessary.
3. LISA and Local G were used for different purpose, I don’t think comparison between this two is needed.
4. The number of CRD may relate with the accessibility to healthcare service in Bangkok metropolitan and some surrounding area. The author may explain whether this point may affect the study result.

I have read this submission. I believe that I have an appropriate level of expertise to confirm that it is of an acceptable scientific standard, however I have significant reservations, as outlined above.

# Response

**Published:** 2018-02-28

**Authors:** Amornrat Luenam

**Affiliations:** Faculty of Public Health, Khon Kaen University, Thailand

**Explanation to question 1.**

We used the SaTScan, using the Poisson probability model and constrained to clusters no larger than 50% of the population at risk and 50% of this study to locate potential circular cluster areas based on the recommendation of Kulldorff M. Kulldorff M (2016) suggested that 50 percent is the default maximum, a smaller maximum may be requested. However, the recommended value is 50 percent (Kulldorff M. 2018, Kulldorff M, Nagarwalla.1995) which reported that a window sized up to 50% of the population at risk can generally reduce negative clusters. Whereas 90%, is more appropriately interpreted as a lower disease rate in the 10% of the area outside the ‘cluster’ rather than as an excess disease rate covering almost the whole study region, therefore, it did not appropriate with our study.

***References:***

Abdurrob A., Kulldorff M. SaTScan Tutoria # 3 Advanced Options. 2016. Available: https://www.satscan.org/tutorials/advancedoptions/SaTScanTutorialAdvancedOptions.pdf

Kulldorff M, 2018. SaTScanTM User Guide for version 9.5

 Kulldorff M, Nagarwalla N. Spatial disease clusters: detection and inference. *Statistics in medicine*. 1995;14(8):799–810.

**Explanation to question 2 and 3**

We conducted Global Moran I, G* test and LISA based on contents and suggestions introduced by Anselin (1995). Specifically, all three tests were undertaken because of the following reasons as clarified in Anselin (1995).

(1) Global Moran I has a limitation. The statistical insignificance of Global Moran I might not indicate that the local association does not exist (Getis and Ord 1992, p. 201). Therefore, the methods of G* and LISA have been developed in order to overcome this incomplete capability of Global Moran I, and both are capable of identifying the local associations.

(2) Based on statistical inference, G* can identify only two cases of localized associations, which are hotspots of high-high and cold spots of low-low. However, LISA has extended this capability, and it can indicate four categories of hotspots, which are high-high, high-low, low-high and low-low. Specifically, LISA can identify both cases of positive spatial correlation (e.g. high-high and low-low) and those of negative spatial association (e.g. high-low and low-high) 

Based on these clarifications, this paper included results obtained from Global Moran I, G* test, and LISA in order to generate the robust results, statically examining spatial associations for both aspects - the global  vs. local and univariate  vs. bivariate. 

***References:***

Anselin, L. Local Indicators of Spatial Association—LISA. *Geographical Analysis*. 1995; 27: 93–115.

Getis, A., and K. Ord. ‘The Analysis of Spatial Association by Use of Distance Statistics.” *Geographical Analysis*. 1992; 24:189-206.

**Explanation to question 4**

We study the CRDs morbidity which is mainly linked to the epidemic of tobacco exposure and indoor and outdoor air pollution in Asian countries (Tan WC, Ng TP, 2008), whereas the mortality may relate with the accessibility to healthcare service in Bangkok metropolitan and some surrounding area.

***References:***

Tan WC, Ng TP. COPD in Asia: where East meets *West. Chest*. 2008; 133(2):517-527.
